# Pharmacological and Non-pharmacological Strategies for Volume Overload in Acute Decompensated Congestive Heart Failure: A Review Article

**DOI:** 10.7759/cureus.6952

**Published:** 2020-02-11

**Authors:** Meer R Zafar, Timothy W Miller, Syed Farrukh Mustafa, Nawfal Al-khafaji

**Affiliations:** 1 Internal Medicine, Sisters of Charity Hospital, Buffalo, USA; 2 Internal Medicine, Jacobs School of Medicine and Biomedical Sciences, Buffalo, USA; 3 Internal Medicine, William Beaumont Hospital, Royal Oak, USA; 4 Cardiology, Trinity Medical Cardiology, Buffalo, USA

**Keywords:** volume overload, acute decompensated heart failure, diuretics, ultrafiltration, renin-angiotensin-aldosterone system, heart failure, diuretic resistance

## Abstract

The epithet of acute decompensated heart failure (ADHF) is volume overload. ADHF is associated with a rising number of hospital admission for volume overload. Medication non-compliance, excessive salt intake, comorbidities, and/or disease progression can attribute to volume overload. Heart failure (HF) therapy has innovated during the past few decades, but diuretics have been the mainstay of treatment. Diuretics are vital even though these drugs stimulate the renin-angiotensin-aldosterone system (RAAS) and lead to adaptive responses like diuretic resistance, neurohormonal activation, and worsening renal function that may be inimical. There has been a thriving interest in cutting-edge strategies to manage volume overload in ADHF. The American College of Cardiology Foundation (ACCF) and the American Heart Association (AHA) guidelines advocate pharmacological and non-pharmacological interventions to treat volume overload in ADHF patients. Ultrafiltration (UF) is, therefore, an emerging stand-in therapy of interest for treating volume overload in ADHF patients. This review article epitomizes available clinical data on the use of diuretics and UF in ADHF patients and identifies challenges for each approach.

## Introduction and background

HF is a complex clinical syndrome due to inability of the heart to adequately fill or eject blood [1]. It is characterized by episodic acute exacerbations which progressively worsen throughout the illness [2]. Based on the Framingham Heart Study, 30-day mortality is around 10%, one-year mortality is 20-30%, and five-year mortality is 45-60% [3]. Since HF is a syndrome and not a disease, its diagnosis relies on clinical examination, which may be challenging [4]. As HF affects more than 5.8 million people in the United States and over 23 million people worldwide it is a public health issue of greatest importance. Its high prevalence and the high cost of treatment represent a considerable burden to the American healthcare system totaling more than $39 billion annually [5]. The expected growing prevalence of HF may be a reflection of increasing incidence, an aging population, improvements in the treatment of acute cardiovascular disease and HF, or a combination of these factors. By 2030, >8 million people in the United States (1 in every 33) will have HF [6]. Approximately 90% of the more than 1 million yearly HF hospitalizations in the United States and Europe are a result of symptoms and signs of volume overload and are associated with readmission rates of 24% and 50% at 30 days and six months, respectively [7]. Recurrent HF-related hospitalizations have uniformly been associated with worse outcomes, independent of age and renal function [8]. Thus identification of modifiable risk factors for primary prevention of HF is critically important for decreasing both incidence and morbidity of HF.

ADHF has been defined as “gradual or rapid change in HF signs and symptoms resulting in a need for urgent therapy.” It is a complex and heterogeneous syndrome characterized by a variable degree of end-organ hypoperfusion and congestion, requiring urgent treatment. It can present as new-onset HF, worsening chronic HF, or advanced HF. Congestion is defined by an abnormally elevated cardiac filling pressure resulting in pulmonary or systemic back up of blood flow, which becomes a major driving force for the symptoms of ADHF. Decongestion is therefore considered a primary goal of acute therapy and is usually achieved in the inpatient setting [9]. Treatment guidelines recommend therapy for patients with HF to be aimed at achieving euvolemia. Testani et al. clearly illustrated in a large cohort that achieving complete decongestion is the most important predictor of good long-term outcomes, with persistent congestion at discharge being the main trigger of readmission [10].

Circulating natriuretic peptide levels are commonly measured in patients with a suspected ADHF episode. Elevated natriuretic peptides levels support the diagnosis of ADHF in patients suffering from dyspnea and many other comorbidities such as valvular heart disease, arrhythmias, pulmonary hypertension, and renal insufficiency [11]. It has been noted that an increased level of natriuretic peptides at discharge for ADHF is associated with a high risk of hospital readmission [12-13].

## Review

Current guidelines advocate the use of intravenous loop diuretics as a first-line agent for the improvement of congestion and symptoms of dyspnea [14]. It is an essential component of current treatment and is administered to approximately 90% of patients who are hospitalized with ADHF. Loop diuretics function by inhibiting the Na+ /K+ /2Cl− in the thick ascending limb of the loop of Henle, resulting in increased excretion of urinary sodium and chloride and subsequent diuresis [15]. Three loop diuretics are currently available: furosemide, bumetanide, and torsemide. Each loop diuretic differs in bioavailability, metabolism, and duration of action. Oral furosemide has a bioavailability of 40-70% while the other loop diuretics: bumetanide and torsemide have bioavailability 80-90%. Another contributing factor to the variable effect of diuretics is the presence of gut edema which often occurs as a result of passive venous congestion seen in HF [16]. Furosemide is also different from bumetanide and torsemide in that furosemide is metabolized in the kidney while the other two loop diuretics are metabolized in the liver. Oral loop diuretics have the same onset of action. However, in their intravenous formulation loop diuretics also differ in their onset. Once administered furosemide usually starts having effects within five minutes, while bumetanide acts sooner in two to three minutes. Currently, there is no intravenous formulation of torsemide to compare. Furosemide has a shortened duration of action of about four to six hours, while torsemide has the longest duration of action of about 12-16 hr. Unlike furosemide and bumetanide, the bioavailability of torsemide remains unchanged with food intake [17].

Common challenges in achieving and maintaining decongestion include inadequate diuretic dosing, diuretic resistance, the “breaking phenomena”, and post diuretic sodium retention also known as the “rebound effect” [17]. Felker et al. found no significant differences in either patient’s global assessment of symptoms or the change in the creatinine level from baseline to 72 hours when diuretic therapy was administered using bolus, as compared to continuous infusion; or with a low-dose strategy, as compared with a high-dose strategy. Felker et al. also noted worsening of renal function had occurred more frequently with the high-dose strategy in the short-term trial; meanwhile, there was no evidence at 60 days of worse clinical outcomes in the high-dose group than in the low-dose group [18].

Prior studies have suggested that high doses of diuretics have a harmful biological effect associated with renal injury. This is thought to occur secondary to electrolyte disturbances or over reactivation of the renin-angiotensin-aldosterone system (RAAS) and the sympathetic nervous systems [19]. Loop diuretics have been shown to up-regulate the RAAS. It promotes aldosterone synthesis and secretion from the adrenal cortex, as well as contributing to ventricular remodeling, myocardial hypertrophy, systemic vasoconstriction, and vascular smooth muscle cell growth. Aldosterone has also been extensively studied and noted to have beneficial cardiac effects as well as some negative systemic effects namely the promotion of inflammation, fibrosis, hypertrophy, and cell death [20-21]. However, these potential adverse effects have been cited as an explanation for observed harmful effects of high doses of loop diuretics. These observations may be subjected to residual confounding bias because high doses of diuretics may be a marker for greater severity of illness rather than a direct mediator of adverse outcomes [18]. Current guideline recommendations state to provide RAAS inhibition with either an angiotensin-converting enzyme inhibitor or angiotensin-receptor blocker in addition to an aldosterone receptor antagonist with loop diuretics in all HF patients with reduced ejection fraction to prevent pathological left ventricular remodeling due to chronic RAAS activation.

Despite preclinical and clinical data supporting the physiological benefits of torsemide, furosemide is the most commonly used loop diuretic [22]. Mentz et al. evaluated the efficacy of torsemide to furosemide in ADHF patients and found that torsemide was associated with a nonsignificant reduction in 30- and 180- day events; torsemide treated patients tended to have features of more severe disease, higher blood urea nitrogen, lower systolic blood pressure, and jugular venous distension [23]. There is conflicting data regarding the beneficial effect of torsemide in inhibition of RAAS, researchers initially noted that torsemide had longer-lasting diuresis and less urinary potassium excretion in animal models [24], and was found to inhibit aldosterone receptor binding in rat kidneys [25], furthermore, torsemide improved clinical markers as noted by a decrease in plasma brain natriuretic peptide and improved echocardiographic measurements of left ventricular function similar to those seen in HF patients receiving spironolactone [26], studies suggesting that torsemide may directly antagonize the cardiac aldosterone receptor on dysfunction myocardial cells that uptake circulating aldosterone.

Diuretic resistance

Diuretic resistance is defined as the inability to reach the desired therapeutic reduction in edema despite the administration of a proper dose of diuretic [27]. With renal insufficiency; this is found to be associated with prolonged hospitalization [28]. Among the proposed mechanisms causing diuretic resistance, the activation of sodium retentive systems as a counter-regulatory response and the changes in loop diuretic pharmacokinetics in HF have received the most attention [29]. The physiology of diuretic resistance; starting with the braking phenomenon, described as an acute reduction in diuretic efficacy with chronic loop diuretic usage. Secondly, the post-diuretic effect refers to increased sodium retention after the loop diuretic has metabolically worn off. Lastly, the rebound effect explains the occurrence of sodium retention when chronic loop diuretic use leads to compensatory increased distal nephron sodium reabsorption [30].

Multiple mechanisms contribute to oral diuretic resistance resulting from bioavailability limitations or altered absorption when taken with food. In the setting of hypoalbuminemia, bioavailability is decreased as charged loop diuretics become protein-bound and are more transported to the kidney while bound to albumin. States of venous congestion and renal vein compression that occur in cardiorenal syndrome may also contribute to diuretic resistance. Similarly, albuminuria is another contributing mechanism of resistance; albumin binds to loop diuretics in the lumen and limits the potential of the free drug to act on the site of action [31].

Currently, there are no specific guideline designs on dealing with diuretic resistance [32]. The Heart Failure Society of America (HFSA), as well as the American College of Cardiology Foundation/American Heart Association (ACCF/AHA), have suggested the following as first-line recommendations for diuretic resistance: sodium and fluid restriction, optimization of the drug regimen, the addition of another diuretic type such as a thiazide which blocks sodium reabsorption at the distal tubule [33], and bolus or continuous intravenous loop diuretic [34]. Eng and Bansal suggest in their case series study the use of a high dose spironolactone diuretic for resistant acute decompensated heart failure [35]. Furthermore, the decreased effective arterial volume is common occurring pathophysiology in heart failure patients as well as cirrhotic patients, which results in the stimulation of RAAS and the sympathetic nervous system. The unnecessary constant stimulation of RAAS causes an increased aldosterone level, which is crucial in the pathogenesis of ascites and loop diuretic resistance. Administration of a natriuretic dose of spironolactone (> 25 mg or equivalent dose) may be an option for immediate relief in patients exhibiting loop diuretic resistant heart failure in addition to close monitoring of electrolytes [36].

Aronson and Burger concluded in their study that loop diuretic dosage is not the sole predictor of urine output. Other factors such as renal function, blood pressure, and fluid intake are also independent predictors of urine output. An elevated right atrial pressure is a marker of fluid overload; which happens to also be an independent predictor of urine output, while the reduced cardiac output is neither. However, net fluid loss is a better predictor of mortality than urine output [37].

Ultrafiltration

Ultrafiltration (UF) is a mechanical modality of sodium and fluid removal, exhibiting adjustable volumes and filtration rates that allow optimal preservation of systemic volume homeostasis. It has re-emerged as an effective strategy for decongestion in ADHF [38]. Previous studies have demonstrated that fluid removal via UF is associated with positive hemodynamic effects. UF increases the arteriovenous renal pressure gradient and decreases neurohumoral activity by reducing central venous pressure without affecting circulating volume due to oncotic pressure, which reabsorbs extravascular volume [39]. The reduction of extravascular lung water with UF allows the rapid improvement of respiratory symptoms such as dyspnea and orthopnea, increased pulmonary gas exchange, better lung mechanics, as well as decreased radiological signs of pulmonary vascular congestion, alveolar and interstitial edema [40,41]. UF also reduces pulmonary artery wedge pressure and increases cardiac output, diuresis, and natriuresis without any changes in heart rate, systolic blood pressure, renal function or electrolyte balance [42]. Since UF also removes a significant amount of total body sodium, it is a crucial contributor to extracellular fluid volume homeostasis. Sodium and its anion are the major determinants of extracellular fluid volume; therefore, total body fluid volume can be reduced more efficiently by UF than by diuretics. Indeed, urine produced by loop diuretics is hypotonic compared to plasma, whereas ultrafiltrate is iso-osmotic and isonatremic [41-42]. Recovery of the renal response to diuretics due to systemic and renal hemodynamic improvement and reversal of the barking phenomenon is a major clinical effect [43]. Despite the strong rationale behind the UF intervention, significant debate remains about which patients, if any, should be treated with it.

Ultrafiltration versus diuretic

The first randomized study that compared intravenous loop diuretics to UF in ADHF was known as RAPID-CHF (Relief for Acutely Fluid-Overloaded Patients with Decompensated Congestive Heart Failure) trial, compared the UF group to the usual diuretic care group. At 24 hours of observation, greater weight loss and fluid removal in the UF group was noted even though an aggressive diuretic dose was given to the usual care group. UF was well-tolerated, no serious complications or significant electrolytes disturbances occurred, neither did any acute renal failure [44]. 

This result simulated the design of the UNLOAD (Ultrafiltration versus Intravenous Diuretics for Patients Hospitalized for Acute Decompensated Heart Failure) trial. Costanzo et al. demonstrated in their study the benefit of early UF in reducing congestion in ADHF with diuretic resistance [45]. In their trial, 60% of high-risk ADHF patients were discharged in less than three days and UF was not associated with worsening of kidney function, electrolyte disturbances or any symptomatic low blood pressure. Also, a sustained drop in plasma brain natriuretic peptide levels were noted along with a significantly decreased rate of rehospitalization during the first three months after initial hospital discharge. Thus, the principal findings support that UF can safely produce greater weight and fluid loss than intravenous diuretics in ADHF patients, along with significant reductions in the rate of hospital admission, rehospitalization and unscheduled medical visits [45].

The first clinical trial to evaluate the biohumoral and hemodynamic effect of UF compared to standard intravenous diuretic therapy in ADHF was The ULTRADISCO (Effects of ULTRAfiltration versus Diuretics on clinical, biohumoral and hemodynamic variables in patients with decompensated heart failure) study. This prospective, randomized study dealt with 15 patients in each group. In the UF group, N-terminal pro-B-type natriuretic peptide levels decreased significantly from 5063+3811 pg/mL at baseline to 1797+1327 pg/mL at post 36 hours (p 0.001). Whereas levels remained essentially unchanged in the diuretic group (6707+3597 pg/mL at baseline to 5271+3251 pg/mL at post 36 hr). Aldosterone levels showed a similar trend; 0.86+1.04 nmol/l at baseline, 0.25+0.23 nmol/l at post 36 in the ultrafiltration group (p 0.001), and 0.73+0.87 to 0.80+0.46 nmol/l at post 36 (p ¼ ns) in the diuretic group. Their findings also showed that the fluid removal obtained with UF in the acute phase had more beneficial effects on hemodynamic variables compared to the diuretic infusion group [46].

There is a conjecture that UF may also remove harmful myocardial depressant factors, perhaps certain cytokines which are known to be deleterious to cardiac function. Investigators have suggested that the removal of cytokines that have predisposing myocardial depressant effects; including tumor necrosis factor-alpha and other inflammatory cytokines, may improve myocardial contractility. In the ULTRADISCO study, It was theorized that the mechanism underlying the improvement of contractility was induced by UF because of lowered cytokine levels [46].

## Conclusions

The most common pathophysiological mechanism underlying both ADHF and the progression of the HF syndrome is volume overload. HF remains the most common cause of hospitalization and the key manifestation is volume overload. Complete decongestion via diuretic strategy is the most important prognosticator of good long-term outcomes and preventing hospitalization. Although there has been clinical data on the effects of diuretic therapy related to reduced intra-arterial volume with RAAS activation, however, no relevance has been rooted between diuretics and cardiovascular mortality. The same is accurate for UF - until substantial clinical evidence is available, its use will be limited to selected cases per current guidelines. New research designed to offset the primary determinants of volume overload could improve the management of patients affected by HF. Until then, diuretic therapy will remain the mainstay of therapy for volume overload in ADHF patients.
